# Conditional-ready mouse embryonic stem cell derived macrophages enable the study of essential genes in macrophage function

**DOI:** 10.1038/srep08908

**Published:** 2015-03-10

**Authors:** A. T. Y. Yeung, C. Hale, J. Xia, P. H. Tate, D. Goulding, J. A. Keane, S. Mukhopadhyay, L. Forrester, O. Billker, W. C. Skarnes, R. E. W. Hancock, G. Dougan

**Affiliations:** 1 Wellcome Trust Sanger Institute, Wellcome Trust Genome Campus, Hinxton, Cambridge, United Kingdom; 2 Centre for Microbial Diseases and Immunity Research, University of British Columbia, Vancouver, BC, Canada; 3 University of Edinburgh/MRC Centre for Regenerative Medicine, Edinburgh, United Kingdom

## Abstract

The ability to differentiate genetically modified mouse embryonic stem (ES) cells into functional macrophages provides a potentially attractive resource to study host-pathogen interactions without the need for animal experimentation. This is particularly useful in instances where the gene of interest is essential and a knockout mouse is not available. Here we differentiated mouse ES cells into macrophages in vitro and showed, through a combination of flow cytometry, microscopic imaging, and RNA-Seq, that ES cell-derived macrophages responded to *S.* Typhimurium, in a comparable manner to mouse bone marrow derived macrophages. We constructed a homozygous mutant mouse ES cell line in the Traf2 gene that is known to play a role in tumour necrosis factor-α signalling but has not been studied for its role in infections or response to Toll-like receptor agonists. Interestingly, *traf2*-deficient macrophages produced reduced levels of inflammatory cytokines in response to lipopolysaccharide (LPS) or flagellin stimulation and exhibited increased susceptibility to *S.* Typhimurium infection.

Macrophages are key immune cells of central importance in microbial recognition and clearance^1^. To date there have been two main approaches to obtain macrophages for in vitro studies, namely the characterization of primary macrophages (often derived from monocytes), or culture of immortalized macrophage-lineage cell lines derived from malignant tumours. Acquisition of primary cells requires access to living organisms, involving ethical challenges and disruptive procedures, as well as issues with the purity of cell populations, limited cell numbers and experimental variation due to the need to use different animals for each experimental series. In addition, primary macrophages are very difficult to genetically manipulate and making them hard to use for studying the impact of mutations in essential genes. Conversely, although immortalized macrophage cell lines are not limited with regards to cell numbers, they are far removed from the normal state^2^.

Stem cells, that retain the ability to self-renew and differentiate, potentially provide a novel alternative approach for studying host-pathogen interactions. Mouse ES cells are derived from the inner cell mass of blastocyst-stage mouse embryos^3^. The pluripotent property of ES cells means that they can be differentiated into diverse cell types, including macrophages. ES cells can also be propagated in large numbers *in vitro* due to their self-renewal property^4^. Moreover, while primary cells are inherently difficult to genetically manipulate, ES cells are highly amenable to genetic manipulation. Therefore, the development of efficient differentiation and conditional gene knock out strategies would provide access to an alternative *in vitro* source of macrophages without some of the above-described disadvantages.

Studies have shown that undifferentiated mouse ES cells lack immunological competence^5^,^6^. In contrast, dendritic cells derived from mouse ES cells display a robust immune signature and can be readily infected by *Salmonella enterica* serovar Typhimurium (*S*. Typhimurium) in a manner that resembles more conventional infection model^7^.

Here we differentiated mouse ES cells into macrophages and showed, through a combination of flow cytometric analysis, microscopy, and transcriptome profiling, that ES cell-derived macrophages (ESDM) responded to *S.* Typhimurium and associated stimuli, including LPS and flagella, in a similar manner to bone marrow derived macrophages (BMDM). We constructed a conditional homozygous mutant mouse ES cell line in the essential Traf2 gene. Using pre-engineered E14 cells expressing site-specific FLP and Cre recombinases in an inducible manner, we modified a previous bi-allelic targeting approach to efficiently generate conditional homozygous mutations. To demonstrate the practicality of using genetically modified ES cells to study the impact of essential genes on macrophage function, we differentiated the Traf2 mutant mouse ES cells to macrophages, and showed that Traf2 played a modulatory role during the differentiation of ES cells into macrophages and a substantial role in the function of these macrophages.

## Results

### Differentiation of murine embryonic stem cells into functional macrophages

**Figure 1:**
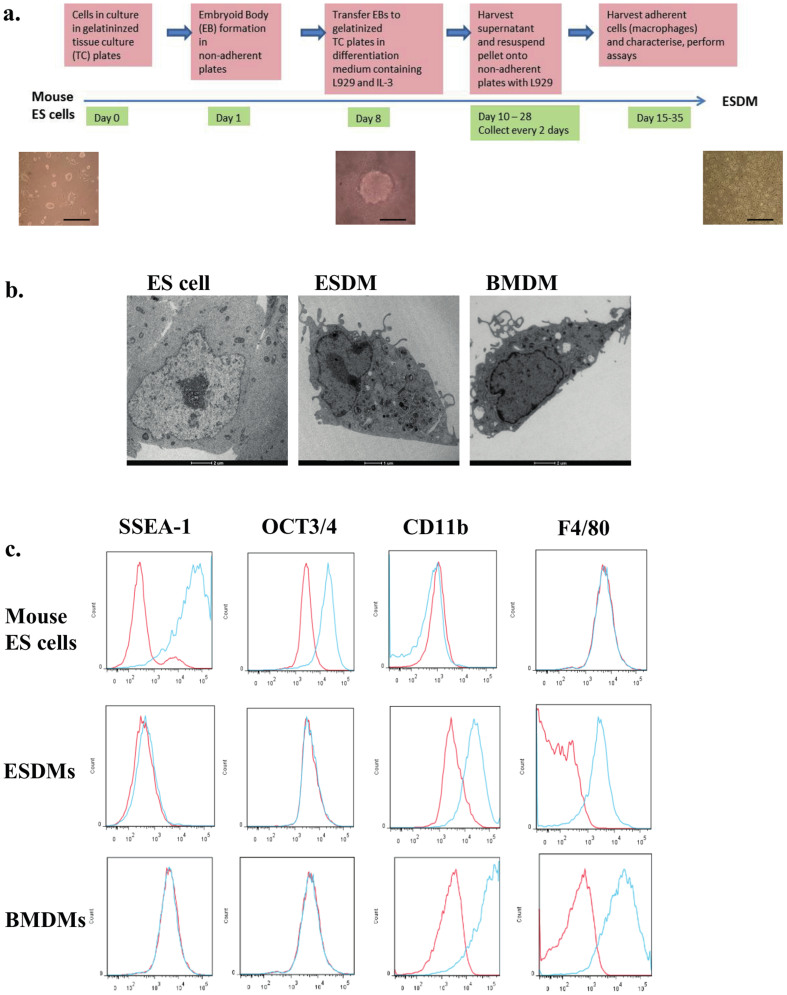
Characteristics of macrophages derived from mouse ES cells. (a) Schematic diagram describing the differentiation procedure from mouse ES cells to macrophages. Phase contrast images under the time line display from left to right images of mouse undifferentiated ES cells, an EB, and ESDM. Scale bars 200 μm. (b) Transmission electron microscopy images of ES cell, ESDM, and BMDM. (c) Flow cytometry analysis of ES cells and ESDM. Red lines represent cells stained with control isotype, blue lines are cells stained with the relevant antibody.

To differentiate mouse ES cells to macrophages, we adapted and modified the approach of Zhuang et al.^8^ (Fig. 1a). Briefly, the procedure involved placing ES cells into 10 cm bacteriological (low-adherence) plates in macrophage differentiation medium (MDM; which lacked the inhibitor of differentiation, LIF, and contained L929 conditioned medium and IL3); this step led to the generation of embryoid bodies (EB). After 6–8 days, the generated EBs in MDM were transferred into gelatinized tissue culture plates to promote adherence of the EB onto the plate and differentiation. Subsequently, supernatants containing non-adherent macrophage progenitors were collected every second day, and adherent macrophages were generated by plating onto low adherence bacteriological plates in MDM; substantial numbers of macrophages appeared after 4 days, with 5 to 10 million macrophages/plate being generated every 2 days from days 14–18. The ESDM were adherent and had long extended plasma projections resembling pseudopodia. Transmission electron microscopy revealed that these ESDM were morphologically indistinguishable from murine BMDMs, exhibiting a large cellular size with a cytoplasm containing vacuoles and organelles and low nuclear to cytoplasm ratios (Fig. 1b). This was in contrast to undifferentiated ES cells that had large nuclei and few organelles. ESDM were characterized by flow cytometry to monitor the expression of stem cell pluripotency and macrophage markers. ESDM displayed markers characteristic of macrophages, including the expression of CD11b, F4/80, CD14 and CD68, while staining negative for the ES cell pluripotency markers Oct3/4 and SSEA-1 (Fig. 1c). Our lab has performed RNA-Seq to compare gene expression of undifferentiated mouse ES cells with that of ESDMs (Yeung ATY and Dougan G, unpublished). The RNA-Seq data revealed upregulation of several macrophage markers, including F4/80, CD11b, CD16, CD64, with fold changes of 1480, 981, 715, 859, respectively in ESDM. Dendritic cell markers CD103 and DEC205 were downregulated with fold changes of −2 and −143, respectively. There were no changes indicated in the RNA-Seq data for expression of CD8 or CD4. Although, we showed that ESDMs expressed low levels of CD11c with a fold change of 2 compared to ES cells, it is known that most macrophages express low to intermediate amounts of CD11c^9^.

To test whether ESDM could recognize and respond to bacterial signals, we stimulated ESDM, BMDM, or undifferentiated ES cells with LPS (agonist of Toll-like receptor, TLR4) flagellin (TLR5 agonist), CpG ODN-1826 (TLR9 agonist), or poly (I: C) (TLR3 agonist) for 4 hours. Stimulated ESDM produced high levels of the pro-inflammatory cytokines and chemokines, TNFα, IP10, KC, MIP1α and MIP1β (Table 1A). Similar responses were observed for BMDM while no detectable levels of any of the tested cytokines or chemokines were produced by undifferentiated ES cells without or with any of the tested agonists (data not shown).

### Phenotypic analysis of ESDM infected with *S.* Typhimurium

**Figure 2:**
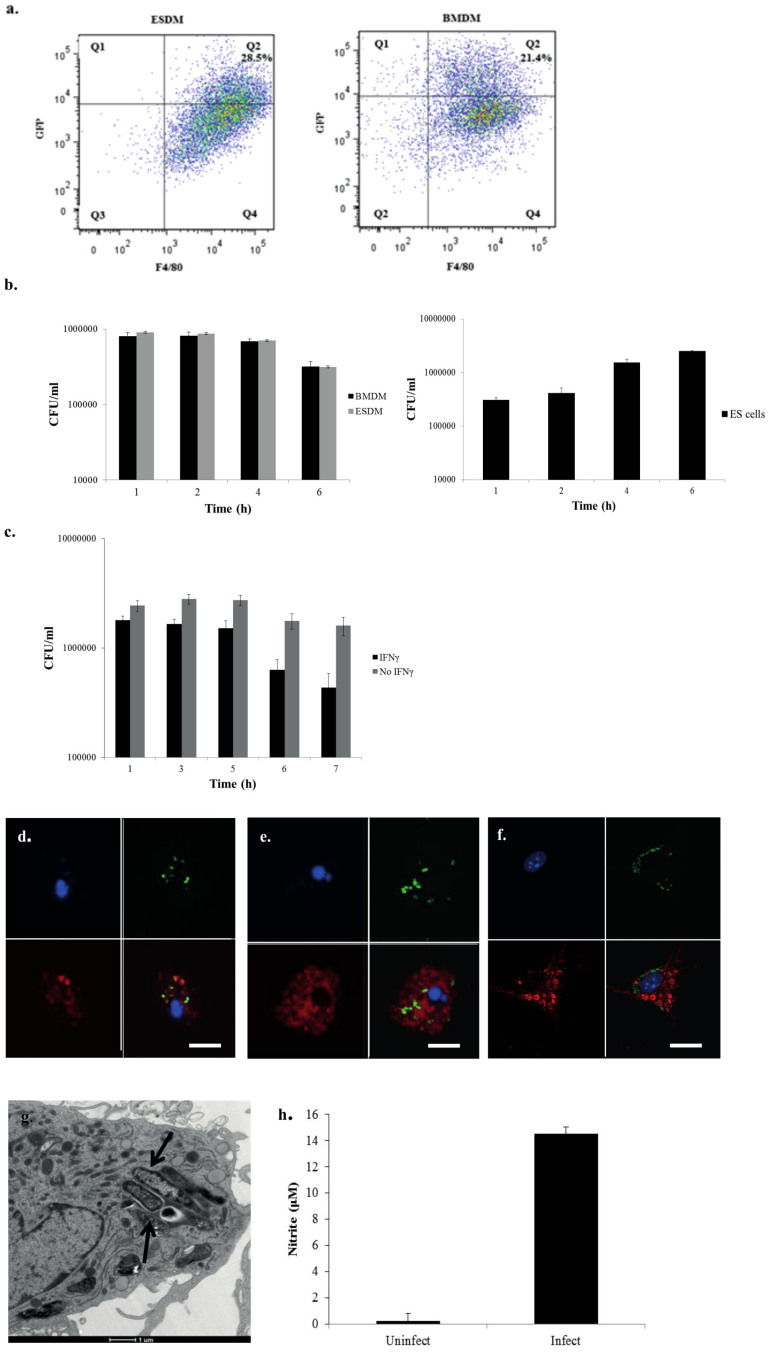
Phenotypic analyses of *S.* Typhimurium SL1344 (pSsaG)-infected ESDM and BMDM. (a) Representative flow cytometry of infected ESDM (left) and BMDM (right), after 4 h post-infection assessing the numbers of cell containing internalized GFP expressing *S.* Typhimurium SL1344 (pSsaG), (b) Bacterial viable cell counts enumerated from untreated ESDM, BMDM (combined left), ES cells (right), (c) IFNγ-pre-treated and un-treated ESDM, infected for various time points by the gentamicin assay (please note that the number of bacterial cells internalized in Fig. 2c was higher by about 3 fold compared to Fig. 2b, due to intrinsic variability and the different cell batches used). Confocal images of ESDM infected with *S.* Typhimurium stained with DAPI (blue) for the ESDM nucleus and either (d) anti-CSA-1 (red), (e) anti-Lamp-1 (red), and (f) caspase staining reagent SR-VAD-FMK. Upper left panels nuclei-stained only, upper right panels GFP- expressing *S.* Typhimurium inside SCV only, lower left CSA-1 (d), Lamp-1 (e) or caspase (f)-stained only, lower right merged images. Scale bars 10 μm. (g) Transmission electron microscopy image of *S.* Typhimurium localized within SCV of an ESDM. Arrows point to SCV. (h) Nitrite production measured in the culture supernatants of uninfected and infected samples of ESDM at 6 h post-infection.

Next we investigated interactions between ESDM and the facultative intracellular enteric pathogen *S.* Typhimurium, using SL1344 (p1C/1, *ssaG*::GFP), which expresses GFP under the control of the *Salmonella* pathogenicity island-2 (SPI-2) -associated promoter *ssaG*, which is activated when *Salmonella* form an intracellular Salmonella Containing Vacuole (SCV). Four hours post challenge, ~28% of the F4/80^+^ ESDM population and 21% of F4/80^+^ BMDM harboured intracellular *S*. Typhimurium (Fig. 2a). Similarly, using a gentamicin resistance assay, ESDMs and BMDMs showed similar numbers of viable bacteria based on colony forming units (CFUs) recovered at 1, 2, 4, and 6 hours post challenge (Fig. 2b). Moreover, the numbers similarly diminished over time, indicating intracellular killing. In contrast, while mouse ES cells also phagocytosed *Salmonella*, but less efficiently, this bacterium was not killed and indeed grew inside ES cells (Fig. 2b). When ESDM were primed overnight with IFNγ, prior to conducting the invasion assay, like normal macrophages they became activated and substantially more efficient at intracellular bacterial killing (Fig. 2c).

Intracellular, pathogenic *Salmonella* avoid phagolysosome fusion in macrophages, and exist within SCVs that resemble late endosomes^10^. Phagocytosed *Salmonella*, as revealed by both GFP fluorescence and antibody staining for *Salmonella* Common Structural Antigen (CSA-1), tended to cluster within macrophages (Fig. 2d). Transmission electron microscopy confirmed the localization of the bacteria within SCVs (Fig. 2g). Although *Salmonella* have developed strategies that modulate the endocytic pathway of macrophages to avoid targeting to the lysosomes, the bacteria recruit Lamp-1 to the SCV^10^. Consistent with this, at 4 h post-infection, the bacteria inside ESDM were predominately co-localized with Lamp-1, as revealed by double staining (Fig. 2e). Furthermore in keeping with the known ability of *Salmonella* infection to induce macrophage apoptosis^11^, staining with the reagent SR-VAD-FMK, showed that *Salmonella*-infected ESDM contained activated caspases (Fig. 2f).

In murine BMDM infected with *Salmonella*, multiple chemokines and pro-inflammatory cytokines are induced, including MIP1α, MIP1β, KC, IP10, and TNFα^12^ (see Supplementary Table S1). No detectable levels of cytokines were found here in undifferentiated ES cells before or after infection with *Salmonella*. In contrast, *S.* Typhimurium-infected ESDM demonstrated strong production of chemokines and cytokines in the supernatants as early as 1 h after infection, and the levels increased over time, eventually matching those of LPS- and flagellin-stimulated ESDM (Table 1B). Similarly at 6 h post-infection both ESDM (Fig. 2h) and BMDM (data not shown), produced significant levels of iNOS, which is known to play a role in controlling *Salmonella* proliferation in macrophages^13^.

### RNA-Seq analysis of ESDM and BMDM infected with *S.* Typhimurium

Phenotypic analyses of ESDM and murine BMDM revealed that these cells shared similar functional properties. To probe this in greater detail, we analysed and compared the transcriptomes of uninfected and *S.* Typhimurium-infected ESDM and BMDM. Gene expression was assessed by RNA-Seq, 4 hr post-infection. Comparing infected to uninfected cells, 3,728 genes were commonly differentially expressed (DE) genes in both ESDM and BMDM [Fold change (FC) of ≥ 2 or ≤ −2 at an adjusted p-value of <0.01] (a full list of DE genes is available on line at http://www.ebi.ac.uk/ena/data/view/ERP002100 and is provided to the reviewers in supplementary and additional material). Up- and down-regulated genes were separately submitted to the innate immunity interactome database and analysis platform InnateDB, and over-representation (OR) analyses carried out. Despite modest differences in culturing methods, unstimulated BMDM and ESDM expressed 12,599 genes in common (read count cut-off of 4), including known macrophage markers, and 93% of these were expressed to a similar level.

**Figure 3:**
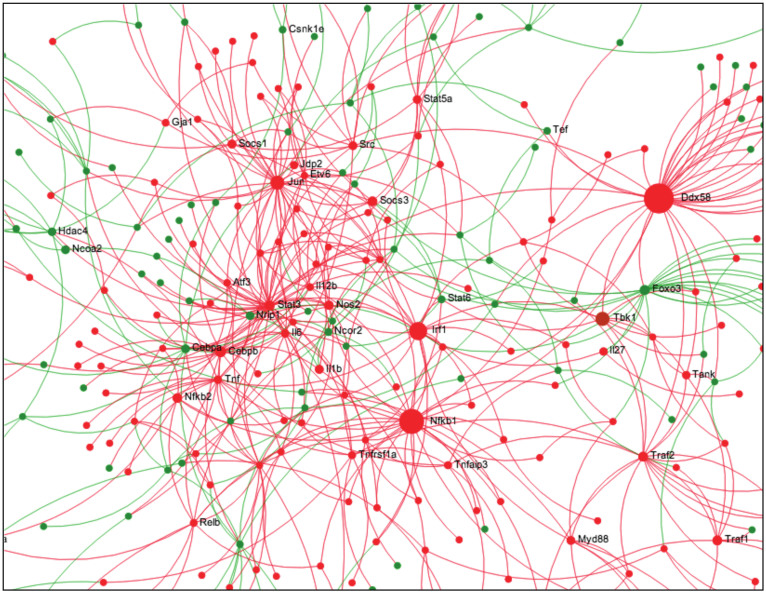
Network visualization of differentially expressed genes between *S.* Typhimurium-infected and uninfected WT ESDM. The program NetworkAnalyst^14^ was applied to the genes dysregulated in infected cf. uninfected ESDM. Below is the largest subnetwork of dysregulated genes with the hub nodes labelled (Hubs are key molecules in signalling since they are highly interconnected; they receive and integrate multiple signals and pass them on to downstream nodes). Shown is a zoomed in picture showing the major hubs. In this figure red nodes were those found to be up regulated and green nodes were downregulated.

Of the 3,728 DE genes shared by both ESDM and BMDM, a total of 73 pathways were found to be significantly over-represented (corrected p-value < 0.05), with 61 up-regulated pathways and 12 down-regulated pathways. More than 90% of the significantly up-regulated pathways were related to innate immunity and/or associated with response to pathogens (see Supplementary Table S2). These pathways included NOD-like receptor signalling, cytosolic DNA-sensing, cytokine and chemokine signalling, MAPK signalling, TLR signalling, IFNγ signalling, Type I interferon signalling, complement signalling, and JAK-STAT signalling. Network analysis^14^ indicated that the major hub proteins in the most highly interconnected network were typical innate immune signalling and effector molecules downstream of Toll-like receptors, including Nfkb1, Nfkb2, Irf1, Myd88, Traf2, Traf1, Cebpb, Socs3, Stat6, Src, Tnf, Il6, etc (Fig. 3).

In accordance with the phenotypes observed after ESDM interaction with *S.* Typhimurium, transcriptomic analysis revealed 1,557 up-regulated genes including strong induction of the inducible NO synthase (Nos2) in infected ESDM (FC = 1,905; for this and all other genes described below similar up-regulation was observed in infected BMDM). Highly elevated expression levels were also observed in infected ESDM for genes encoding cytokines including IL1α (FC = 1461), IL1β (FC = 544), IL6 (FC = 700), IL12β (FC = 877), and chemokines including, CCL3 (MIP1α) (FC = 232), CCL4 (MIP1β) (FC = 568), CXCL9 (MIG) (FC = 488), and CXCL10 (IP10) (FC = 271). Anti-inflammatory IL10, which plays a role in controlling the inflammatory response during *Salmonella* infection was also up-regulated (FC = 72.4). *Salmonella* infection is known to elevate macrophage expression of surface receptors involved in communication with other cells of the immune system. Here, we observed up-regulation of CD40 (FC = 288), mannose receptor 2 (Mrc2) (FC = 9.8), CD86 (FC = 72), and major histocompatibility complex (MHC) class II genes H2-Aa (FC = 211) and H2-Ab1 (FC = 33.5). In addition, there was up-regulation, in infected ESDM, of genes involved in apoptosis including Casp1 (FC = 6.5), Casp4 (FC = 22.8), Casp7 (FC = 2.6), and Malt1 (FC = 23). Interestingly, our RNA-Seq experiment revealed significant up-regulation in infected ESDM and BMDM of 3 genes that we recently found to be critical in murine *Salmonella* infections, namely Rassf1 (FC = 2.5), Dusp1 (FC = 10), and Trex1 (FC = 9.7) (https://www.sanger.ac.uk/mouseportal/).

Of the 2171 genes down-regulated upon *Salmonella* infection in ESDM and BMDM, 12 significantly over-represented pathways were identified. The majority of these pathways were associated with energy metabolism, including fatty acid β-oxidation, glycolysis and branched chain amino acid catabolism which might play a role in Salmonella infection and/or control of intracellular growth. Moreover, gene ontology (GO) term over-representation analysis, using Innate DB, revealed significant down-regulation of other cellular processes during macrophage infection related to cell proliferation, including cell cycle, cell division, and DNA replication. Genes that were dysregulated in only one of the two cell types tended to have low read counts and did not tend to cluster into pathways suggesting that these reflect stochastic variations and/or experimental differences in their derivation.

### Macrophages derived from an inducible *traf2*^-/-^ mouse ES knockout cell line

To further illustrate the attractive potential of using macrophages differentiated from ES cells to study macrophage function, we explored the potential use of these cells to uncover novel functions of host genes for which mutations induce a lethal phenotype in homozygous mice. Our RNA-Seq study showed that the *traf2* gene was significantly up-regulated in infected ESDM and BMDM (FC = 3.1). TNF receptor associated factor 2 (Traf2) has been reported to play an important role in inflammatory processes, programmed cell death, and interferon responses due to its ability to be activated by TNFα^15^. It is part of a family of proteins that function as adaptor molecules for TNF superfamily members by associating with the intracellular domain of these receptors to mediate downstream signalling events such as NFκB and AP-1 activation. It is a major hub protein and interacts with between 359 and 512 different proteins based on experimentally verified and orthology-predicted interactions in the curated database InnateDB. These include a variety of direct binders that have been studied in detail, including CD27, CD30, CD40, CD137, TNFR2, receptor activator of nuclear factor-κB (RANK), and cellular inhibitor of apoptosis protein-1 (cIAP-1)^16^,^17^,^18^,^19^. Intriguingly however a role in innate immunity and bacterial infection has not been described.

The frequency of breeding viable *traf2^-/-^* mice is low and dependent on the genetic background, e.g. only 6.5 to 10% of *traf2^-/-^* offspring were viable in one study^15^. In addition viable *traf2^-/-^* mice showed very poor survival and succumbed soon after birth^15^, as indeed confirmed by us (https://www.sanger.ac.uk/mouseportal/). Consequently, to study the function of Traf2 in various cell types in a *traf2^-/-^* mutant would require obtaining cells derived from embryos or from very young mice^15^,^20^. Other groups have generated *traf2*-deficient mutants *in vitro* by transfecting cells lines with Traf2 siRNA^21^,^22^, although the intrinsically inflammatory nature of siRNA, incomplete knockdown and potential off-target effects make this approach less than ideal. Interestingly, it has been suggested that Traf2 is required for human macrophage differentiation^22^. Here, we generated both heterozygous and homozygous mouse ES cells harbouring mutations in *traf2* and differentiated these lines into macrophages.

### Differentiation of mouse *traf2*^-/-^ ES cells to macrophages

**Figure 4:**
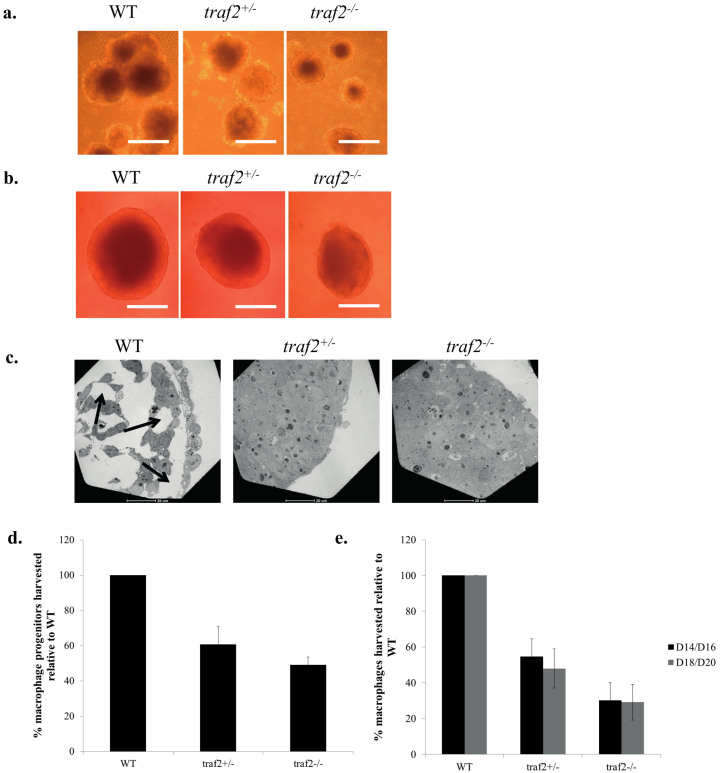
Differentiation of mouse WT, heterozygous *traf2^+/−^*, and homozygous *traf2^-/-^* ES cells to macrophages. (a, b) Light microscopy images of WT, *traf2^+/−^*, and *traf2^-/-^* EB grown on (a) 10 cm bacteriological dishes (scale bars 200 μm) and (b) a 96- well low adherence plate. Scale bars 100 μm. (c) Transmission electron microscopy images of cavity formation in day 8 EB. Arrows point to cavities formed. (d, e) Graphical representations for the percentage of (d) macrophage progenitors and (e) macrophages recovered for the *traf2* mutants compared to the WT. From days 14–20, one dish of 6 × 10^5^ WT mES cells typically generated between 5–10 × 10^6^ ESDMs.

Wild type (WT), *traf2^+/-^*, and *traf2^-/-^* ES cells were grown for several passages; no differences in morphology or numbers of cells were observed. Next, we differentiated WT, heterozygous and homozygous *traf2* mutant ES cells into macrophages using our differentiation procedure (Fig. 1; Methods). Interestingly, after day 5, the sizes of the EBs for the *traf2* mutant line were visibly smaller than those of the WT (Fig. 4a). As EB generated in the 10 cm bacteriological plates are generally variable in size, we grew EB in the wells of round bottomed 96-well low adherence plates. The sizes of *traf^-/-^* EB (area ~50% of WT) were significantly smaller than those of *traf2^+/-^* (area ~75% of WT) which themselves were smaller than WT EB (Fig. 4b). Subsequently, we transferred the EB onto gelatinized dishes to generate floating macrophage precursors. The number of precursor cells collected from the *traf2^-/-^* plates was overall 50% less than those of the WT (Fig. 4d) and we subsequently recovered only 30–40% as many *traf2^-/-^* macrophages (ESDM), when compared to the WT (Fig. 4e). Western blot studies confirmed the absence of expression of the TRAF2 protein in the *traf2^-/-^* mutant macrophages, while flow cytometry, confirmed the presence of macrophage-specific markers, CD11b, F4/80, CD14 and CD68, and the loss of stem cell pluripotency markers Oct3/4 and SSEA-1 (Supplementary Fig. S1).

To investigate a possible explanation for the decreased number of *traf2^-/-^* ESDM generated from the macrophage differentiation procedure, we investigated the WT and mutants at the EB stage, and observed abnormal cavitation in the *traf2^-/-^* and *traf2^+/^*^-^ mutants cf. WT. Cavitation is a morphogenetic process that transforms the solid embryonic ectoderm into a columnar epithelium surrounding a cavity^23^. Maturation of EB structures recapitulate formation of endoderm on the surface of the inner cell mass and formation of a central cavity in the inner ectodermal cell core. Interestingly transmission electron microscopy of *traf2^-/-^*, *traf2^+/-^*, and WT EB revealed striking differences in this property (Fig. 4c). While we observed substantial cavitation in day-8 WT EB, the day-8 *traf2^-/-^* and *traf2^+/-^* EB demonstrated no cavitation. Leaving the *traf2* mutant EB beyond 8 days slightly increased their sizes, but they still lacked cavitation, even though WT EB had completed their cavitation process (data not shown). The lack of cavitation observed for *traf2*-deficient EB, even with heterozygous cells was consistent with the possibility that *traf2*-deficient EB lack a step in the early differentiation process when compared to WT EB, and that a full gene dosage of *traf2* is required to promote normal EB formation. Furthermore these data show that this process, phenotypically observed as a deficiency in cavitation, did not block the subsequent differentiation of EBs into macrophages. Instead, these mutant EBs generated fewer macrophage progenitors when compared to WT EB.

### Phenotypic analysis of *traf2*^-/-^ ESDM

**Figure 5:**
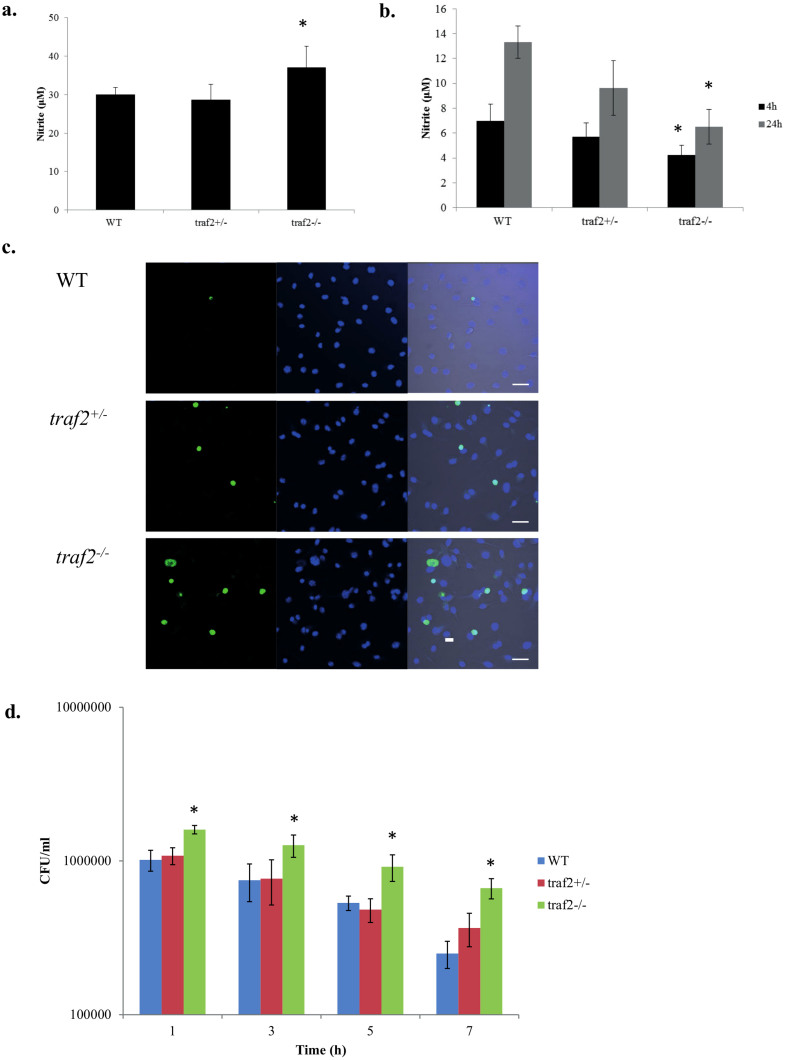
Responses to TNFα and LPS stimulation, and to *S.* Typhimurium infection of WT, *traf2^+/−^*, and *traf2^-/-^* ESDM. Production of nitrite in culture supernatants after (a) 24 h TNFα stimulation, (b) 4 and 24 h LPS stimulation. (c) Cells were labelled by TUNEL to detect apoptotic cells (green in left panels) and stained with DAPI to detect nuclei (blue in middle panels). Right panels merged images. Scale bars 40 μm. (d) Bacterial cell counts enumerated from *S.* Typhimurium-infected ESDM at various time points. * represent statistically significant difference (*P* < 0.05) between WT and *traf2^-/-^* mutant as determined using two-way ANOVA. Equal numbers of WT and mutant cells were seeded for the various stimulation experiments.

Although significantly lower numbers of ESDM were generated for the *traf2^-/-^* when compared to the WT, we nevertheless managed to harvest 3 to 5 × 10^6^ *traf2^-/-^* ESDM per dish every 2 days enabling a full suite of experiments. We first investigated whether *traf2^-/-^* ESDM behaved similarly to *traf2^-/-^* macrophages obtained from *traf2*-deficient mice. As reported using macrophages from *traf2*-deficient mice^22^, *traf2^-/-^* ESDM produced significantly elevated levels of nitrite (Fig. 5a) and pro-inflammatory cytokines TNFα (182% of WT) and IP10 (152% of WT), in response to TNFα stimulation (Table 1B). Moreover, as expected^23^, we observed that *traf2^-/-^* ESDM exhibited increased sensitivity to TNFα-induced apoptosis (Fig. 5b and 5c).

Some Traf family members, e.g. Traf3 and Traf6, have been documented to play roles in TLR pathways^24^, but no such role has been shown for Traf2. Upon stimulation with LPS or flagellin for 4 h, it was observed that *traf2*^-/-^ ESDM produced significantly lower levels of pro-inflammatory cytokines when compared to WT ESDM (Table 1B). Thus Traf2 contributed to both the TLR4 and TLR5 signalling pathways. In contrast, the effect on TLR-agonist-induced cytokines was opposite to that previously observed for TNFα-induced cytokines (as confirmed here; Table 1B). Conversely *traf2^+/-^* ESDM showed minor to intermediate changes in these properties despite the above-mentioned differences in EB cavitation and yield of ESDM, indicating that this difference was not merely due to altered maturation. Moreover, in *S.* Typhimurium infection studies, *traf2^-/-^* ESDM exhibited enhanced uptake of the bacteria at 1 h post-infection and a reduced ability to eliminate intracellular bacteria (Fig. 5d). These investigations of *traf2^-/-^* ESDM have therefore identified a new role for this TRAF family member.

## Discussion

The exploitation of mouse ES cells to elucidate gene function in normal biological and disease processes has opened up tremendous new possibilities in scientific research. Unlike many other cell lines, ES cells are normal diploid cells with virtually unlimited proliferative abilities and have the capacity to differentiate into many different cell types *in vitro*. Tremendous progress has been made in defining culture conditions to promote differentiation of mouse ES cells along various specific lineages^8^,^25^,^26^. Coupled with the success in achieving complete gene inactivation in ES cells, valuable information can now be gained on gene function. This is particularly powerful in cases where a homozygous knockout mouse is embryonic lethal or debilitated to the extent that any phenotype might relate to the condition of the mouse rather than a particular treatment model. Moreover, the ability to generate differentiated ES cells in high purity and numbers is useful for a wide range of phenotypic and genomic studies, including transcriptomics, proteomics, and epigenetic profiling.

To validate using ESDM in place of BMDM, we showed by transmission electron microscopy that ESDM were indistinguishable in morphology and size from BMDMs. Flow cytometry revealed that ESDM had lost ES cell markers and had gained macrophage markers. In contrast, there was limited overlap in genes expressed in undifferentiated ES cells and ESDM (Yeung ATY and Dougan G, unpublished). It is important to note that here we compared ESDMs to BMDMs since the latter are derived in an analogous manner from progenitor cells in the mouse and represent the most common model for studying ex vivo macrophage biology. Nevertheless there are other models that could have been used such as primary cultured macrophages or peripheral blood monocyte-derived macrophages, and macrophages are known to differentiate into a spectrum of macrophage phenotypes, and we cannot extrapolate our results to these situations.

We also investigated the applicability of using ESDM to study host-pathogen interactions *in vitro* by treating ESDM with a series of TLR agonists to reveal significant up-regulation of various inflammatory cytokines and chemokines. Similar to BMDM, ESDM were capable of internalizing the facultative intracellular pathogenic bacterium *S.* Typhimurium. Transmission electron microscopy studies revealed phagocytosed *S.* Typhimurium became internalized within a SCV. Moreover, while *S.* Typhimurium mediated recruitment of Lamp-1 to the SCV, there was limited fusion with lysosomes, a phenomenon similarly observed for *Salmonella*-infected BMDM. Comparison of transcriptome profiles of ESDM and BMDM by RNA Seq after infection with *S.* Typhimurium revealed a major overlap with a total of 3,728 commonly affected genes. A substantial proportion of these were involved in host defences and immune recognition, signalling pathways and responses. Taken together our data indicate that ESDM represent functional macrophages that behave similarly to primary macrophages.

A number of gene inactivation strategies have been developed over the years for mammalian cells, including RNA interference (RNAi), gene trapping and gene targeting^27^,^28^,^29^. A major caveat for the first two strategies is that both of these methods depend on genes/transcripts expressed in mouse ES cells. These strategies are also limited in their ability to generate targeted gene modifications. For example RNAi leads to incomplete knockdown and off-target effects; also there is a tendency for nucleic acids to induce inflammatory pathways through TLR3,7/8 and 9 and intracellular nucleic acid pattern recognition receptors. Conversely in gene trapping, the integration of the cassette occurs randomly so there is no control as to where exactly in the gene structure the disruption occurs. Gene targeting, on the other hand, can be used to engineer virtually any alteration in the mammalian genome, ranging from point mutations to large chromosomal rearrangements, by homologous recombination^28^. Using a serial targeting strategy and/or Cas9-Crispr methods^30^, bi-allelic mutations of a target gene in ES cells can be efficiently generated.

Traf2 is a complex protein with more than 450 interactors in human cells (www.innatedb.ca). Several studies^15^,^20^, including our own, have confirmed that homozygous *traf2* knockout mice die after about 2–3 weeks, making study of this genetic mutant challenging, since investigating very young mice and accommodating the influence of systemic morbidity and/or immunosuppressive treatments make interpretation of any results difficult. To address this, we constructed heterozygous and homozygous *traf2* mutant ES cells and differentiated them into macrophages. The homozygous mutant exhibited the largest changes in cytokine production in response to TNFα and various TLR agonists (Table 1B). The response to TNFα, which has been described before^15^,^20^, included a 50% increase in IP10 and a 82% increase in TNFα. The response to flagellin and LPS, which had not been studied previously, was more complex. There were decreases of between 50% and 90% in M1 cytokines/chemokines IP10, TNFα, MIP1α, MIP1β, but a 2–3 fold increase in M2a chemokine KC. Interestingly the lack of cavitation in EBs derived from heterozygous and homozygous *traf2* mutant ES cells did not prevent differentiation into macrophages, and other mutant ES cells that we have made (e.g. *irak1^-^*) had no cavitation defect nor decrease in resultant macrophage numbers.

To date, the function, in host-pathogen interactions, of a large proportion of genes in the mouse genome still remain to be elucidated, or are difficult to study due to essentiality. While the use of whole animal models to study this great number of candidate genes would not be feasible, mutant ES libraries provide a potential method for elucidating the functions of these genes. Recently, Koike-Yusa et al. developed a technology using a comprehensive mouse lentiviral CRISPR gRNA library to generate genome-wide homozygous mutant mouse ES cell libraries^30^. Using the methods established here, one could potentially differentiate the mutant ES cells from the library into specific lineages of interest to study and identify novel host factors involved in host-pathogen interaction.

## Methods

All experiments were approved by and carried out in accordance with the Wellcome Trust Sanger Institute approved guidelines.

### Bacterial strains and culture conditions

*S. enterica* serovar Typhimurium SL1344 is a murine-virulent *Salmonella* strain, whereas SL1344 (p1C/1, *ssaG*::GFP) is a derivative that directs the expression of green fluorescent protein (GFP) from the *ssaG* promoter that becomes activated once a *Salmonella* containing vacuole (SCV) is formed within host cells^31^. Bacteria were routinely grown on Luria-Bertani agar (L agar) plates with the appropriate antibiotic for selection. Antibiotics were added at the following concentrations: ampicillin, 100 μg/ml or kanamycin, 50 μg/ml.

### ES cell line and culture condition

The mouse E14 iCreiFlp ES cell line (derived from the 129P2 mouse strain) was routinely cultured in 0.1% gelatin-treated flasks under 5% CO2 at 37°C in complete Glasgow's minimal essential medium (GMEM) (Sigma) supplemented with 2 mM glutamine (Invitrogen), 1 mM sodium pyruvate (Invitrogen), 1X nonessential amino acids (Invitrogen), 10% (v/v) fetal bovine serum (FBS), a 1:1000 dilution of a β-mercaptoethanol stock solution (14.3 M, Sigma), and 1000 U/ml leukemia-inhibiting factor (LIF) (Chemicon).

### Construction of a homozygous conditional *traf2* mouse ES cell line.

To generate inducible homozygous knockout lines of mouse ES cells we engineered E14 cells, derived from the 129P2 mouse strain (Wellcome Trust Sanger Institute) and having a quite stable genome, to express doxycycline-inducible FLPo and tamoxifen-inducible Cre recombinases (S. Wormald, W. Zhang and W.C. Skarnes, unpublished). Using these E14 iCreiFlp cells, we disrupted both alleles of the target gene by serial targeting^32^ to generate cells that carried a conditional and a deletion allele. In choosing the exon and surrounding region to flox, we were careful to avoid small RNA and other conserved elements. Targeting the first allele of each gene used available knockout-first targeting constructs from the EUCOMM/KOMP resource^33^. To delete the second allele, EUCOMM/KOMP intermediate vectors were modified by exchanging the selectable marker to blastocidin resistance and deleting the floxed exon in Cre-expressing bacteria. Following application of tamoxifen, the floxed exon was completely eliminated by the action of Cre recombinase based on qPCR. A schematic of the strategy is shown in Supplementary Fig. S2.

To induce gene inactivation by tamoxifen treatment, growth medium for the inducible homozygous *traf2* mutant ES cells was replaced with medium containing 0.4 μM 4-hydroxy-tamoxifen (4-OHT, Sigma). Controls included treatment of wild type (WT) and heterozygous *traf2* mutant with 4-OHT, and treatment of the same clones with media containing an equivalent volume of 99% ethanol (solvent used to prepare 4-OHT stock). The cells were grown for 48 h and screened for loss of the floxed critical exon via Loss-of Allele (LOA) assay. The confirmed clones were subsequently expanded and frozen for stocks. After several passages, we confirmed complete loss of Traf2 protein in the tamoxifen-treated homozygous mutant ES cells by Western immunoblot. ES cells were analysed by flow cytometry for pluripotent stem cell markers to ensure there were no changes in pluripotency after the tamoxifen treatment.

### Differentiation of ES cells into macrophage progenitors

Mouse ES cells were differentiated into monocytes as previously described^8^. To form EB, ES cells were seeded at 6 × 10^5^ cells/9 cm bacteriological dish (90 mm Sterilin 101Vr20 triple vent) in 20 ml of macrophage differentiation medium (MDM) for 6-8 days. MDM is similar to complete Glucose Minimal Essential Medium (GMEM; Gibco) with the exception that the differentiation inhibitor Leukemia Inhibitory Factor (LIF) was excluded from the medium and the medium was supplemented with 15% L929 conditioned medium and 1 ng/ml IL-3 (RnD). To obtained conditioned medium, L929 fibroblasts were cultured in Dulbecco's minimal essential medium (DMEM) (Sigma) supplemented with 10% FBS (Sigma), 2 mM glutamine and 1% penicillin/streptomycin (Sigma). Three days after they had reached confluence, conditioned medium was harvested from adherent L929 cells, filtered through a 0.22 μm membrane and stored in aliquots at −20°C. Six to 8 days old EB were transferred onto gelatin-treated tissue culture dishes (100 mm TC treated corning 430167, 150 mm TC treated Corning 430599). Starting on day 4, after transfer, supernatants of adherent EB containing floating macrophage progenitors were collected and plated onto bacteriological low adherence dishes; medium was re-added to the adherent EB plates and the process repeated every 2 days.

### Differentiation of macrophage progenitors into ESDM

Macrophage progenitors from the supernatant of adherent EB dishes, after plating onto bacteriological low adherence dishes, were cultured for up to 7 days to form an adherent macrophage monolayer on a 10 cm bacteriological dish with 20 ml of RPMI-1640 medium supplemented with 2 mM glutamine, 10% heat-inactivated FBS and 15% L929 conditioned medium. The adherent ESDMs were harvested by adding lidocaine solution (4 mg/ml lidocaine-HCl containing 5 mM EDTA in Dulbecco's phosphate-buffered saline and gentle scraping using a Cell Lifter.

### Preparation of BMDM from mice

Mice (129 line, Wellcome Trust Sanger Institute) of 5–10 weeks old were sacrificed by cervical dislocation. The care and use of mice were in accordance with the UK Home Office regulations (UK Animals Scientific Procedures Act 1986). Bone marrow was obtained by flushing the tibia and femurs with PBS. The cells were centrifuged at 1,100 rpm for 5 min, at 4°C and the resultant pellet re-suspended with RPMI-1640 medium supplemented with 15% L929 conditioned medium, 10% heat-inactivated FBS, 2 mM glutamine and 1% penicillin/streptomycin and transferred to a Petri dish. The macrophage progenitors were cultured for a week to allow the cells to attach and divide until there was a confluent monolayer of macrophages on the dish.

### Invasion assays of ES cells and macrophages with *S.* Typhimurium

Mouse ES cells or macrophages (ESDM or BMDM) were seeded into either 6-well plates (2.5 × 10^5^ cells/well for ESC, 1 × 10^6^ cells/ml for ESDM or BMDM) or 24-well plates (0.5 × 10^5^ cells/well for ESC, 1 × 10^6^ cells/well for ESDM or BMDM) and cultured for 1–2 days. *S.* Typhimurium was grown at 37°C shaking for 4.5 h and then diluted 1:50 in L broth and grown at 37°C overnight as a static culture to optimize *Salmonella* pathogenicity island 1 (SPI-1) gene expression. The bacterial cultures were diluted in RPMI-1640 (Sigma) containing 10% (v/v) heat-inactivated FBS (Sigma) and 2 mM glutamine to obtain a multiplicity of infection (MOI) of 50. After 30 min of incubation, cells were washed with phosphate-buffered saline (PBS) and gentamicin (50 μg/ml) was added to kill extracellular bacteria. Cells were incubated for the indicated length of time and lysed with 1% Triton X-100. Dilutions of the cell lysates were plated onto L agar plates to determine the number of intracellular bacteria colony forming units (CFU).

### Confocal microscopy

Cells grown on coverslips were washed and fixed with 4% paraformaldehyde, permeabilized with 0.1% Triton X-100 and stained with various antibodies, including antibody to *Salmonella* Common Structural Antigen (CSA-1) conjugated to tetramethylrhodamine (TRITC) (KPL), Lysosome Associated Membrane Protein 1 (LAMP-1) (AbCam), or SR-VAD-FMK FLICA poly caspase reagent (Invitrogen) followed by counterstaining with relevant conjugated anti-species secondary antibodies. Stained coverslips were washed and mounted onto slides with ProLong Gold containing DAPI (Invitrogen). The preparations were visualized with an LSM510 META confocal microscope (Zeiss).

### Transmission electron microscopy

For transmission electron microscopy, cells were fixed in their culture wells with a mixture of 2.5% glutaraldehyde and 4% paraformaldehyde on ice for 1 h. Cells were rinsed with 0.1 M sodium cacodylate buffer (pH 7.42), scraped and pelleted. The pellet was fixed in 1% osmium tetroxide at room temperature for 1 h, followed by 1% buffered tannic acid for 30 min and then a 1% aqueous sodium sulphate rinse for 10 min. The sample was dehydrated in an ethanol-propylene oxide series with 2% uranyl acetate added at the 30% step and embedded in Eponaraldite for 24 h at 60°C. Ultrathin sections (60 nm) were cut on a Leica EMUC6 ultramicrotome, contrasted with uranyl acetate and lead citrate, and imaged on an FEI 120 kV Spirit Biotwin with a Tietz F415 digital TemCam.

### Flow cytometry

For flow cytometry, cells were lifted off from the surface of culture wells (trypsin for mouse ES cells and lidocaine for ESDM and BMDM), fixed with 1% paraformaldehyde and incubated with specific conjugated antibodies, including F4/80, CD11b, SSEA-1, Oct3/4, CD68 and CD14. For the intracellular markers, cells were permeabilized with saponin buffer. Isotype matched antibodies conjugated to the same fluorophores were used as negative controls. Samples were analysed using the FACSAria II (BD Biosciences).

### Cytokine and chemokine secretion

Mouse ES cells, ESDM and BMDM were separately incubated with various TLR agonists, including lipopolysaccharide (LPS) from *S. enterica* (100 ng/ml; Sigma), flagellin from *S.* Typhimurium (1 μg/ml; Invivogen), CpG oligodeoxynucleotides (CpG ODN, 10 μg/ml; Invivogen), or polyinosinic: polycytidylic acid (polyIC, 5 μg/ml; Invivogen) for 4 h. Culture supernatants were harvested, sterile filtered and frozen at −80°C until assayed. Mouse tumor necrosis factor α (TNFα), interleukin 6 (IL6), IL1α, IL1β, interferon γ-induced protein (IP10), keratinocyte chemoattractant (KC), macrophage inflammatory protein (MIP)1α, MIP1β were measured using a Luminex FLEXMAP 3D system (Millipore). Data analysis was carried out using the xPONENT Multiplex Assay Analysis software (Millipore).

### RNA-Seq and data analysis

ESDM and BMDM were separately infected or not with *S.* Typhimurium for 30 min, and then cells were washed and gentamicin-treated to kill any remaining extracellular bacteria and prevent further infection. Gene expression was measured by RNA-Seq after 4 h post-infection. Total RNA from 4 separate studies (biological repeats) of uninfected and *S.* Typhimurium infected ESDM and BMDM were isolated using the RNeasy Mini Kit (Qiagen). The total RNA quantity was assessed by Spectrophotometer NanoDrop-1000 v3.1.0 (Thermo Scientific) and the RNA quality was analysed using the 2100 Bioanalyzer (Agilent), following the manufacturer's instructions. Total RNA was converted into libraries of double stranded cDNA using the Illumina TruSeq RNA Sample Preparation v2 Kit. mRNA was purified from 5 μg of total RNA using an oligo dT magnetic bead pull down. Fragmentation was carried out using metal ion-catalyzed hydrolysis. A random-primed cDNA library was synthesized and the resulting double-stranded cDNA was used as the input for library preparation. Overhangs were repaired with a combination of fill-in reactions and exonuclease activity to produce blunt ends. Adenylation of blunt ends was followed by ligation to Illumina Pair-end Sequencing adapters containing unique index sequences. cDNA enrichment was carried out by 10 cycles of PCR amplification. Samples were quantified and pooled based on post-PCR analyses with an Agilent Bioanalyzer. Pools were size-selected using the LabChip XT Caliper. The multiplexed library was then sequenced on the Illumina HiSeq 2000, 75 bp paired-end read length. The range of total reads per biological sample was from 34 to 165.2 million reads of which 77–84% could be mapped to version mm10 of the mouse reference genome.

Each lane of Illumina sequence was assessed for quality based on GC content, average base quality and Illumina adapter contamination. The Burrows-Wheeler Aligner alignment tool^34^ version 0.6.1-r104 was used to align the reads to the reference mouse genome version mm10. Gene expression values (RPKM) were computed from the read alignments to the coding sequencing using an in-house script. Differentially expressed genes were calculated by the DESeq package^35^ in R software using two-fold change [log_2_(fold-change)] and adjusted p-value < 0.05 (cut-off at 5% false discovery rate (FDR)) as the threshold. Analyses of infected vs. uninfected cells, revealed 6,387 and 5,579 differentially expressed (DE) genes, for ESDM and BMDM respectively, with a fold change (FC) ≥ 2 or ≤ −2 at an adjusted p-value of <0.01 (Accession number ERP002100 at http://www.ebi.ac.uk/ena/data/view/ERP002100). Of these 3728 were commonly dysregulated and many genes that were not found to be commonly dysregulated had very low sequence counts, and might well have been non-active transcripts.

InnateDB (www.innatedb.ca) was used to perform cellular Pathways enrichment using the over-representation analysis option that examines overrepresentation of pathway genes (relative to their overall abundance). The settings for the analysis algorithm (Hypergeometric) and the multiple testing correction method (The Benjamin & Hochberg correction for the false discovery rate) were used. Only pathways that were significantly up- or down- regulated with a p-value < 0.05 were reported here. RNA-Seq data can be accessed under ERP002100 at http://www.ebi.ac.uk/ena/data/view/ERP002100.

### Statistics

Statistical significance was performed with GraphPad Prism software. Student's *t* test was used for 2-group comparisons, and ANOVA was used for comparisons involving 3 or more groups.

## Author Contributions

R.E.W.H., G.D., A.T.Y.Y., C.H., L.F., S.M. and O.B. conceived and designed the experiments. A.T.Y.Y., C.H., R.E.W.H., P.H.T., D.G. and W.C.S. performed the experiments. A.T.Y.Y., J.A.X., J.K. and C.H. analysed the data. A.T.Y.Y. and R.E.W.H. wrote the manuscript. All authors reviewed the manuscript.

## Supplementary Material

Supplementary Information Dataset 1

## Figures and Tables

**Table 1:** Concentrations of cytokines and chemokines produced by ESDM: A. WT ESDM: After 4 h stimulation with various TLR agonists, or at various times during infection with *S.* Typhimurium SL1344 (p1C/1, *ssaG*::GFP). B. Comparison of WT, *traf2^−/+^*, and *traf2^-/-^* ESDM. Results were obtained after 24 h stimulation with TNFα, or 4 h stimulation with LPS or flagellin. Each value in parts A and B represents the average of at least 3 independent measurements ± standard deviation. Equal numbers of WT and mutant cells were seeded for the various stimulation experiments

			Cytokines and Chemokines Induction (pg/ml)				
Stimulus	Time	Cell Type	IP10	TNFα	KC	MIP1α	MIP1β
**A.**							
LPS	**4 h**	WT	1201 ± 460	620 ± 196	1562 ± 389	5133 ± 602	8445 ± 808
Flagellin	**4 h**	WT	656 ± 244	607 ± 180	1731 ± 264	3915 ± 809	6221 ± 714
CpG ODN	**4 h**	WT	725 ± 312	198 ± 28	321 ± 124	1097 ± 567	5934 ± 670
Poly-IC	**4 h**	WT	1389 ± 431	43 ± 13	100 ± 15	1038 ± 218	1915 ± 105
*Salmonella*	**1 h**	WT	26 ± 6	76 ± 13	241 ± 55	69 ± 19	71 ± 22
*Salmonella*	**2 h**	WT	119 ± 54	160 ± 52	719 ± 69	263 ± 89	381 ± 76
*Salmonella*	**4 h**	WT	893 ± 28	487 ± 70	1996 ± 231	1416 ± 110	2342 ± 332
*Salmonella*	**6 h**	WT	1373 ± 110	735 ± 89	2883 ± 452	3203 ± 219	7832 ± 987
**B.**							
LPS	**4 h**	WT	1055 ± 224	619 ± 248	1547 ± 619	4907 ± 1472	8052 ± 1240
		*traf2^−/+^*	756 ± 213	550 ± 102	2122 ± 1412	3395 ± 1133	7639 ± 890
		*traf2^-/-^*	582 ± 131*	155 ± 78*	4771 ± 1400*	1694 ± 900*	4089 ± 524*
Flagellin	**4 h**	WT	701 ± 79	665 ± 120	2017 ± 334	4113 ± 1808	7390 ± 2450
		*traf2^−/+^*	449 ± 124	758 ± 113	2921 ± 400*	2576 ± 942	4521 ± 1156
		*traf2^-/-^*	64 ± 24*	303 ± 68*	3116 ± 399*	773 ± 831*	1031 ± 1000*
TNFα	**4 h**	WT	5008 ± 725	3104 ± 519	-	-	-
		*traf2^−/+^*	5615 ± 790	3222 ± 600	-	-	-
		*traf2^-/-^*	7596 ± 890*	5637 ± 714*	-	-	-

Values are shown as net cytokine/chemokine production, which was calculated by subtracting the value for the unstimulated control. Each value represents the average of at least 3 measurements ± standard deviation. "-" means not tested *Indicates a significant difference (*P* < 0.05) between mutant and WT.
