# Supplementary material for: Conditional-ready mouse embryonic stem cell derived macrophages enable the study of essential genes in macrophage function
Source: Sci Rep. 2015 Mar 10;5:8908. doi: 10.1038/srep08908 (PMC4354151; doi:10.1038/srep08908)
Supplement: Supplementary Information — Dataset 1 [file srep08908-s1.doc]

**Supplementary Figures and Tables**

**Conditional-ready mouse embryonic stem cell derived macrophages enable the study of essential genes in macrophage function**

A.T.Y. Yeung, C. Hale, J. Xia, P.H. Tate, D. Goulding, J.A. Keane, S. Mukhopadhyay, L. Forrester, O. Billker, W.C. Skarnes, R.E.W. Hancock, & G. Dougan

**Supplementary Fig. S1:** Various assays to confirm loss of *traf2* expression and expression of macrophage markers in *traf2^-/-^* ESDMs. a) Loss of allele assay, b) RT-qPCR, c) western blot, and d) flow cytometry.

**Supplementary Fig. S2.** Schematic diagram for generation of conditional homozygous mutant by serial targeting.

**Supplementary Table S1:** Concentrations of cytokines and chemokines produced by BMDM after 4 h stimulation with various TLR agonists or infection with *S.* Typhimurium SL1344 (p1C/1, *ssaG*::GFP).

| **Stimulus** | **Cytokines and Chemokines Induction (pg/ml)** | | | |  |
| --- | --- | --- | --- | --- | --- |
|  | **IP10** | **TNFα** | **KC** | **MIP1α** | **MIP1β** |
| LPS | 1586 ± 351 | 1133 ± 95 | 2249 ± 233 | 6204 ± 700 | 9038 ± 765 |
| Flagellin | 1006 ± 292 | 873 ± 25 | 1865 ± 201 | 5585 ± 410 | 6800 ± 567 |
| CpG ODN | 744 ± 231 | 462 ± 88 | 279 ± 69 | 3806 ± 229 | 6535 ± 502 |
| Poly-IC | 1852 ± 459 | 366 ± 72 | 154 ± 56 | 3382 ± 250 | 4629 ± 296 |
| *Salmonella* | 772 ± 152 | 445 ± 21 | 1334 ± 82 | 1249 ± 125 | 2399 ± 300 |

Values are shown as net cytokine/chemokine production, which was calculated by subtracting the value for the unstimulated control.

**Supplementary Table S2:** Innate DB over-representation pathways by ESDMs and BMDMs at 4 hours post-infection.

| **A. Upregulated** | | |  |  |
| --- | --- | --- | --- | --- |
|  |  | |  |  |
| **Pathway Name** | | **Source Name** | **Ratio of pathway uploaded gene count/genes in InnateDB for this entity** | **Pathway p-value (corrected)** |
| Cytokine-cytokine receptor interaction | | KEGG | 31% | 7.50E-24 |
| NOD-like receptor signaling pathway | | KEGG | 39% | 8.75479E-09 |
| Toll-like receptor signaling pathway | | KEGG | 30% | 7.60581E-08 |
| Leishmaniasis | | KEGG | 36% | 9.44341E-08 |
| Osteoclast differentiation | | KEGG | 28% | 9.71785E-08 |
| Cytosolic DNA-sensing pathway | | KEGG | 38% | 9.43957E-08 |
| Jak-STAT signaling pathway | | KEGG | 24% | 4.84601E-07 |
| Type I diabetes mellitus | | KEGG | 36% | 6.79339E-07 |
| Malaria | | KEGG | 37% | 6.25423E-07 |
| MAPK signaling pathway | | KEGG | 19% | 1.09827E-06 |
| Focal adhesion | | KEGG | 21% | 2.52601E-06 |
| Amoebiasis | | KEGG | 25% | 2.70963E-06 |
| Hepatitis C | | KEGG | 23% | 2.9964E-06 |
| Toxoplasmosis | | KEGG | 24% | 3.06027E-06 |
| ECM-receptor interaction | | KEGG | 28% | 3.11973E-06 |
| Chemokine signaling pathway | | KEGG | 21% | 5.233E-06 |
| African trypanosomiasis | | KEGG | 45% | 5.22713E-06 |
| Allograft rejection | | KEGG | 35% | 6.13183E-06 |
| Graft-versus-host disease | | KEGG | 33% | 1.12205E-05 |
| Interferon gamma signaling | | REACTOME | 44% | 1.48148E-05 |
| Chagas disease (American trypanosomiasis) | | KEGG | 24% | 5.48686E-05 |
| RIG-I-like receptor signaling pathway | | KEGG | 28% | 5.61731E-05 |
| Viral myocarditis | | KEGG | 25% | 8.90613E-05 |
| JAK STAT pathway and regulation | | INOH | 18% | 0.000127568 |
| Antigen processing and presentation | | KEGG | 26% | 0.000152325 |
| RIP-mediated NFkB activation via DAI | | REACTOME | 53% | 0.000163401 |
| TNFR1 signaling pathway | | INOH | 40% | 0.000297812 |
| Interleukin-1 processing | | REACTOME | 83% | 0.000375425 |
| RIG-I/MDA5 mediated induction of IFN-alpha/beta pathways | | REACTOME | 67% | 0.000368778 |
| Intestinal immune network for IgA production | | KEGG | 31% | 0.000392656 |
| Cell adhesion molecules (CAMs) | | KEGG | 19% | 0.000397235 |
| Integrin signaling pathway | | INOH | 23% | 0.000476127 |
| Phagosome | | KEGG | 18% | 0.000574739 |
| Staphylococcus aureus infection | | KEGG | 28% | 0.000600453 |
| TRAF6 mediated IRF7 activation | | REACTOME | 60% | 0.000708834 |
| Immunoregulatory interactions between a Lymphoid and a non-Lymphoid cell | | REACTOME | 24% | 0.001664346 |
| Apoptosis | | KEGG | 22% | 0.001941263 |
| CD28 co-stimulation | | REACTOME | 63% | 0.002231699 |
| STING mediated induction of type 1 IFN | | REACTOME | 50% | 0.002410912 |
| Autoimmune thyroid disease | | KEGG | 23% | 0.002519214 |
| IL-23 signaling | | INOH | 100% | 0.006244054 |
| c-src mediated regulation of Cx43 function and closure of gap junctions | | REACTOME | 100% | 0.006244054 |
| TRAF6 mediated NF-kB activation | | REACTOME | 43% | 0.006124408 |
| TAK1 activates NFkB by phosphorylation and activation of IKKs complex | | REACTOME | 50% | 0.007501701 |
| Pathways in cancer | | KEGG | 14% | 0.008196397 |
| Hypertrophic cardiomyopathy (HCM) | | KEGG | 20% | 0.008439501 |
| Growth hormone receptor signaling | | REACTOME | 35% | 0.008262042 |
| Regulation of Complement cascade | | REACTOME | 40% | 0.008490381 |
| Chemokine receptors bind chemokines | | REACTOME | 31% | 0.009034711 |
| Hematopoietic cell lineage | | KEGG | 20% | 0.00964121 |
| IL-1 signaling pathway | | INOH | 26% | 0.010250922 |
| GPCR signaling | | INOH | 14% | 0.011026099 |
| FCGR activation | | REACTOME | 38% | 0.011425044 |
| Small cell lung cancer | | KEGG | 19% | 0.011706693 |
| Interleukin-1 signaling | | REACTOME | 25% | 0.011725581 |
| Regulation of IFNG signaling | | REACTOME | 42% | 0.016033155 |
| Integrin cell surface interactions | | REACTOME | 21% | 0.016725074 |
| Alternative complement activation | | REACTOME | 75% | 0.01693545 |
| Destabilization of mRNA by AUF1 (hnRNP D0) | | REACTOME | 21% | 0.018920326 |
| Dissolution of Fibrin Clot | | REACTOME | 50% | 0.018401632 |
| Inactivation of Cdc42 and Rac | | REACTOME | 50% | 0.018401632 |
| Signaling by Robo receptor | | REACTOME | 50% | 0.018401632 |
| TRAF3-dependent IRF activation pathway | | REACTOME | 50% | 0.018401632 |
| p38MAPK events | | REACTOME | 50% | 0.018401632 |
| Collagen biosynthesis and modifying enzymes | | REACTOME | 33% | 0.018631029 |
| Dilated cardiomyopathy | | KEGG | 18% | 0.020242166 |
| ISG15 antiviral mechanism | | REACTOME | 32% | 0.024542063 |
| Glycosphingolipid biosynthesis - lacto and neolacto series | | KEGG | 28% | 0.024318862 |
| ER-Phagosome pathway | | REACTOME | 20% | 0.025295647 |
| Adipocytokine signaling pathway | | KEGG | 19% | 0.026255089 |
| Complement and coagulation cascades | | KEGG | 18% | 0.026547017 |
| HS-GAG biosynthesis | | REACTOME | 30% | 0.030313957 |
| Constitutive Signaling by NOTCH1 t(7;9)(NOTCH1:M1580 K2555) Translocation Mutant | | REACTOME | 60% | 0.03108226 |
| Extrinsic Pathway | | REACTOME | 60% | 0.03108226 |
| Generation of second messenger molecules | | REACTOME | 29% | 0.037860994 |
| Prion diseases | | KEGG | 23% | 0.044294627 |
| Activation of NF-kappaB in B Cells | | REACTOME | 19% | 0.049050406 |
| **B. Downregulated** | |  |  |  |
|  | |  |  |  |
| **Pathway Name** | | **Source Name** | **Ratio of pathway uploaded gene count/genes in InnateDB for this entity** | **Pathway p-value (corrected)** |
| Peroxisome | | KEGG | 30% | 1.03017E-05 |
| Metabolic pathways | | KEGG | 13% | 5.6267E-06 |
| Propanoate metabolism | | KEGG | 39% | 0.000607017 |
| Fanconi Anemia pathway | | REACTOME | 44% | 0.005999799 |
| Glycogen breakdown (glycogenolysis) | | REACTOME | 55% | 0.010635244 |
| Valine, leucine and isoleucine degradation | | KEGG | 27% | 0.012050022 |
| Beta-oxidation of pristanoyl-CoA | | REACTOME | 63% | 0.012734663 |
| Non-small cell lung cancer | | KEGG | 24% | 0.025636422 |
| Thiamine metabolism | | KEGG | 100% | 0.030711024 |
| Role of phospholipids in phagocytosis | | REACTOME | 37% | 0.028556977 |
| Phosphatidylinositol signaling system | | KEGG | 20% | 0.049068839 |
| Cysteine and methionine metabolism | | KEGG | 27% | 0.049601613 |
| Lysosome | | KEGG | 17% | 0.046581082 |
